# Novel approaches for quantitative electrogram analysis for intraprocedural guidance for catheter ablation: A case of a patient with persistent atrial fibrillation

**DOI:** 10.15761/NMBI.1000121

**Published:** 2017-06-16

**Authors:** SP Arunachalam, S Kapa, SK Mulpuru, PA Friedman, EG Tolkacheva

**Affiliations:** 1 Department of Biomedical Engineering, University of Minnesota, Minneapolis, MN, USA; 2 Department of Cardiovascular Medicine, Mayo Clinic, Rochester, MN, USA; 3 Department of Cardiovascular Medicine, Mayo Clinic, Phoenix, AZ, USA

**Keywords:** Atrial fibrillation, rotor, pivot point, ablation, multiscale entropy, multiscale frequency, kurtosis, recurrence period density entropy and empirical mode decomposition

## Abstract

**Purpose:**

Atrial fibrillation (AF) is the most common sustained cardiac arrhythmia that causes stroke affecting more than 2.3 million people in the US and is increasing in prevalence due to ageing population causing a new global epidemic. Catheter ablation with pulmonary vein isolation (PVI) to terminate AF is successful for paroxysmal AF but suffers limitations with persistent AF patients as current mapping methods cannot identify AF active substrates outside of PVI region. Recent evidences in the mechanistic understating of AF pathophysiology suggest that ectopic activity, localized re-entrant circuit with fibrillatory propagation and multiple circuit re-entries may all be involved in human AF. The authors developed novel electrogram analysis methods and validated using optical mapping data from isolated rabbit hearts to accurately identify rotor pivot points. The purpose of this study was to assess the feasibility of generating patient-specific 3D maps for intraprocedural guidance for catheter ablation using intracardiac electrograms from a persistent AF patient using novel electrogram analysis methods.

**Methods:**

A persistent AF patient with clinical appointment for AF ablation was recruited for this study with IRB approval. 1055 electrograms throughout the left and right atrium were obtained for offline analysis with the novel approaches such as multiscale entropy, multiscale frequency, recurrence period density entropy, kurtosis and empirical mode decomposition to generate patient specific 3D maps. 3D Shannon Entropy, Renyi Entropy and Dominant frequency maps were also generated for comparison purposes along with local activation time and complex fractionated electrogram analysis maps.

**Results:**

Patient specific 3D maps were obtained for each of the different approach. The 3D maps indicate potential active sites outside the PVI region. However, presence of rotors cannot be confirmed and validation of these approaches is required on a larger dataset.

**Conclusions:**

Conventional catheter mapping system can be used for generating patient specific 3D maps with short time series analysis using the novel approaches.

## Introduction

AF is the most common sustained cardiac arrhythmia that is associated with increased risk of stroke, heart failure and death affecting more than 2.3 million people in the United States and over 30 million people worldwide [1]. Pharmacological therapy for treating AF is only sub-optimal and also causes serious side effects [2]. Catheter ablation to treat paroxysmal AF has been shown up to 87% successful using pulmonary vein (PV) isolation [3–8]. However, in patients with persistent AF ablation is challenging since the location of triggers is unclear and it has been shown that triggers commonly arise outside the PVs. Recent research suggests that AF ablation has a success rate of 28% with 51% after multiple repeat procedures in persistent AF [9].

While there is still debate about AF cause and maintenance, many investigators argue that spatially localized rotors maintain AF which can be present in both atria [10]. Ablation of rotor pivot points has been suggested to terminate AF in paroxysmal, persistent and long-standing AF patients [11,12]. The presence of rotors in AF patients is usually confirmed using activation patterns, although several mapping technologies have been developed, such as using dominant frequency (DF) and complex fractionated electrogram analysis (CFAE) [9]. There are several challenges in these current mapping technologies due to various noises, misleading phase and activation times that distort data, and clinical signals that may not faithfully represent local activation, etc. A recent, review of the current quantitative electrogram analysis methods revealed several novel approaches for identifying active sites for cardiac arrhythmias [13]. However, none of these methods have proven efficacy in accurately identifying rotor pivot points as suitable sites for catheter ablation to terminate AF or other arrhythmias.

Rotors are caused by reentrant mechanisms which are known to be responsible for maintaining AF. They are composed of a complex rotor pivot zone surrounded by a stable peripheral zone. Identification of the rotor pivot zone as a suitable ablation target has been the research focus for many investigators. However, these investigations are challenged with short time series data in the clinical setting. In the clinical setting electrogram recordings are frequently limited to 2.5–5 second segments due to the need for frequent catheter repositioning during the procedure, challenging conventional mapping approaches to precisely identifying substrates in AF and other arrhythmias. Several researchers have attempted clinical trials using DF, CFAE and phase mapping and more recently a novel approach called spatial temporal dispersion.

Reduction in the left-to-right DF gradient was demonstrated following ablation of sites with high DF and a reduced risk of atrial arrhythmia recurrence [14]. However, only 11% termination of AF was seen in persistent AF compared with 72% for paroxysmal AF patients. Another study reported a termination of AF for only persistent AF patients out of 30 who were enrolled for DF ablation [15]. The largest study to assess DF ablation to date, the RADAR-AF trial, randomized 232 patients to strategies of high DF site ablation and/or conventional PVI [16]. In persistent AF patients, freedom from atrial arrhythmia was seen in 67% of patients who underwent PVI plus high DF site ablation, similar to the 63% of patients who underwent PVI alone [16]. These results indicate the inefficacy of DF approach to guide catheter based AF ablation for persistent AF patients. However, it has been shown that the DF for rotor is uniform throughout its spatial locations demonstrating its inability identify the core of the rotor [17].

Clinical results with CFAE ablation have demonstrated mixed results. Initial studies showed 95% terminating of persistent AF with no recurrence, but other groups were not able to reproduce the results [18–22]. In general no advantage was seen with CFAE lesions delivered to paroxysmal AF patients with some degree of improvements in persistent AF patients in maintaining sinus rhythm according to meta-analysis of CFAE ablation [23]. A more recent STAR-AF 2 trial with randomized 589 persistent AF patients to PVI, PVI plus CFAE ablation, or PVI plus linear ablation in the atrium showed no benefit with CFAE [24]. Moreover, different groups use customized algorithms for calculating CFAE scores for ablation in their patient groups which makes it difficult for reproducibility in other sites. Nevertheless, it appears that CFAE ablation should be attempted with other novel strategies than can provide new directions for clinical guidance which would need validation with more clinical trials.

Narayan et al used a phase mapping approach known as focal impulse and rotor modulation (FIRM), in their FIRM trial to terminate AF in persistent AF patients [25]. However, one criticism is that the signal processing approaches taken for phase mapping was not disclosed contradicted by non-reproducibility of this approach by other groups, However, the authors demonstrated mapping of rotors in humans using FIRM [26]. The authors also demonstrated 86% termination of AF using FIRM guided ablation in persistent AF patients [27–29]. However, another independent group who used FIRM guided ablation was not able to reproduce these results in a blinded study. More recent study demonstrated discrepancy with low SE and DF in FIRM indicated target sites [30]. These results suggest the need for more rigorous validation of this approach, given the challenge that exists with the Hilbert transform operation to achieve Hilbert phase from EGMs.

Recently a novel approach called spatiotemporal dispersion approach was used to terminate AF in patient groups. The idea behind this approach is that, electrograms recorded simultaneously by a multipolar catheter displaying both spatial and temporal dispersion areas are indicative of AF drivers, regardless of whether these electrograms are fractionated or not [31]. 105 AF patients were prospectively enrolled in the study and the authors tagged and ablated only regions displaying electrogram dispersion during AF which resulted in 95% success rate in terminating AF. The authors validated the spatiotemporal dispersion of EGMs near the driver source using numerical simulations and optical mapping in ex vivo ovine atrium. The results offer huge promise, which however need further validation from several groups.

We, recently used Shannon entropy approach to obtain 3D SE maps which demonstrated higher regions of SE near the right atrial appendage outside the PV region for a persistent AF patient [17]. However, with optical mapping data, lower SE values were observed at the rotor core compared to the periphery which challenges the investigation of rotors using intracardiac electrogram using SE. Recently, obtaining patient-specific computational models are gaining popularity to locate rotors that may maintain AF. However, these methods are complex, and may challenge intraprocedural guidance for AF ablation [32]. With a clear need for a robust spatio-temporal rotor mapping technique, the authors reported several novel quantitative electrogram analysis techniques suitable for short time series analysis such as intracardiac electrogram analysis namely multiscale entropy (MSE), multiscale frequency (MSF), kurtosis, recurrence period density entropy (RPDE) and empirical mode decomposition (EMD) [33–38]. Each of these approaches was validated using optical mapping data from isolated ex vivo rabbit hearts for accurate identification of rotor pivot points. The purpose of this work was to (i) demonstrate the feasibility of generating patient specific 3D maps using these novel approaches that can potentially provide intraprocedural guidance during catheter ablation for AF and (ii) confirm the presence of active sites outside the PVI region for a persistent AF patient in accordance with the current hypothesis for AF maintenance.

## Materials and methods

### Recruitment of a persistent AF patient

The informed consent with IRB approval from a 64 year old woman with persistent symptomatic atrial fibrillation was obtained who was recommended for ablation after failing antiarrhythmic drugs including dofetilide. The patient was brought to the cardiac catheterization electrophysiology lab in the fasting state for the ablation procedure.

### Intracardiac electrogram data collection

The electrophysiology study was performed in the Cardiac Catheterization Laboratory, in the Department of Cardiovascular Medicine in Mayo Clinic, Rochester, MN USA. The patient had transesophageal evaluation to exclude atrial thrombus before the procedure. Electrophysiological study was performed in the post absorptive state under general anesthesia. The LA was accessed transeptally, and a single bolus of 100 IU/kg heparin was administered and repeated to maintain activated clotting time above 190 sec. The electrograms were collected during AF and the electrophysiologist was able to obtain a complete right and left atrial map during AF using the PentaRay^®^ NAV catheter.

Electro-anatomic mapping (CARTO, Biosense-Webster) was performed prior to AF ablation. The CARTO mapping system has a sensor position accuracy of 0.8 mm and 5°. With the Thermacool SF catheter, the 3D geometry of the chamber was reconstructed in real time, and at each point, the system records the 12-lead ECG and bipolar electrograms sampled at 1000 Hz and low pass filtered at 30 to 500 Hz, thus allowing the electrophysiological information to be color coded and superimposed on the anatomic map. Evenly distributed points were recorded using a fill threshold of 20 mm throughout the RA, LA, and CS. At each point, 5–15 s electrograms together with the surface ECG were acquired. Endocardial contact during point acquisition was facilitated by fluoroscopic visualization of catheter motion, the distance to geometry signaled by the catheter icon on the CARTO system, and confirmed in a subset with intracardiac echocardiography.

During ablation, full pulmonary vein isolation was completed with wide area circumferential ablation around both sets of pulmonary veins. A cavotricuspid isthmus (CTI) line was also done given a clinical history of CTI-dependent flutter. The ablation procedure with PVI successfully terminated the AF and the patient was maintained on dofetilide for 3 months post ablation per standard of care post-ablation and then this was discontinued. Over 9 months follow-up since discontinuing dofetilide, she had one paroxysm of symptomatic AF in the setting of receiving anesthesia for orthopedic surgery but otherwise no known recurrence. The study data was stored in Mayo Clinic’s protected database and the entire study was exported in .xml format for offline analysis following the IRB guidelines for offline analysis.

### Intracardiac electrogram data collection

The data export only allowed 2.5 s of electrogram data and with a sampling rate of approximately 1000 Hz, time series data with 2500 sample points for each electrogram were available for analysis with the novel approaches. A total of 1055 electrograms were obtained from this patient with 642 electrograms in the LA and 413 electrograms in the RA. The export data also contained the electroanatomic mapping information, catheter coordinates, the locations from which the electrogram were obtained etc. which are useful for recreating the 3D geometry to generate patient-specific 3D maps with offline analysis.

The electrograms were processed using MSE, MSF, kurtosis, RPDE and EMD approaches along with SE, Renyi Entropy and DF to obtain eight different 3D maps for inferences and to compare the results from each approach. Briefly, MSE approach calculates nearest neighbour time averaged time series with a scale factor of ‘3’ and quantifies the repetitiveness of the time series in linear space [33]. MSF approach uses eight log-normal filter banks with cut off frequencies 0.625, 1.25, 2.5, 5, 10, 20, 40 and 80 Hz and MSF index is obtained as the average of the sum of the ratios of the filter outputs [34–35]. Kurtosis approach quantifies the ‘peaknedness’ of the probability density function of the time series data [36]. RPDE approach quantifies the repetitiveness of the time series data in phase space by transforming the data into phase space with time delay of ‘2’ in three dimensions. A small sphere of radius ‘ε’ is defined for each time point, and every time the time series returns to this sphere, the recurrence time is calculated and entropy of the probability density function of this recurrence time yields RPDE [37]. Finally, the time series is decomposed using EMD approach into its intrinsic mode functions (IMF). The complexity of IMF is quantified using modified multiscale entropy using the 2nd, 3rd and 4th IMF’s [38]. MATLAB software was used to implementing these quantitative electrogram analysis methods. The 3D locations from where the electrograms were acquired were used to generate patient specific 3D maps by superimposing on the electroanatomic map using custom MATLAB.

**Figure 1:**
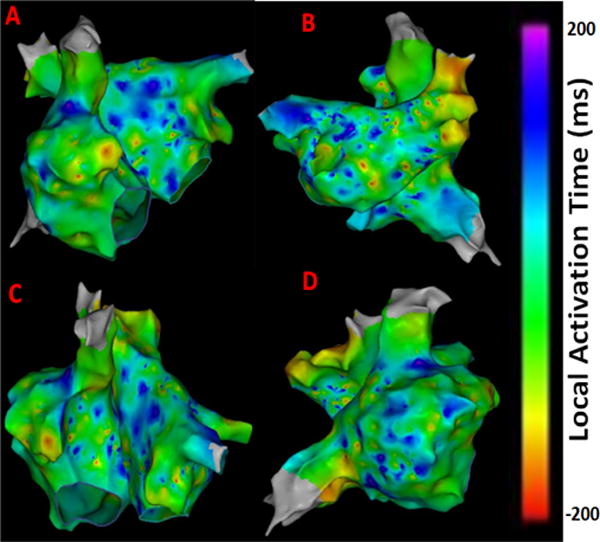
LAT maps from the persistent AF patient. (A) Antero-Posterior view; (B) Postero-Anterior view; (C) Left Anterior Oblique (LAO) view; (D) Right Anterior Oblique (RAO) view.

LAT maps were obtained from the CARTO system shown in Figure 1 for comparing the results from the novel approaches reported in this work. Bluish to purple regions indicate low activation times that may correspond to chaotic active sites. Greenish to yellow regions represent higher activation times that may correspond to normal atrial activity. As seen from Figure 1 late activity can be seen near right atrial appendage (RAA), around right sided pulmonary veins and anterior regions of the inferior vena cava (IVC) indicating potential areas of active AF sites. The electrograms obtained from these regions are expected to portray abnormal atrial activation which can be captured by the novel quantitative electrogram analysis showing higher complexity.

**Figure 2:**
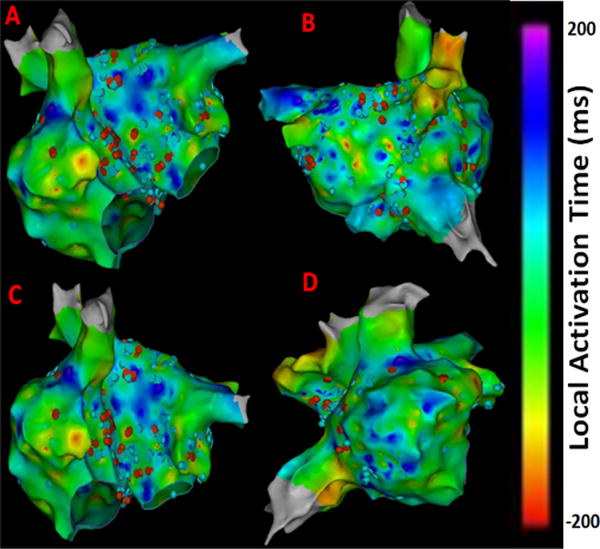
CFAE points superimposed on LAT maps from the persistent AF patient. (A) Antero-Posterior view; (B) Postero-Anterior view; (C) Left Anterior Oblique (LAO) view; (D) Right Anterior Oblique (RAO) view. Red dots have >8 peaks, blue dots fit within the 5–8 peak range. Electrograms having interval confidence level (ICL) score ≥ 5 were considered CFE positive by CARTO.

**Figure 3:**
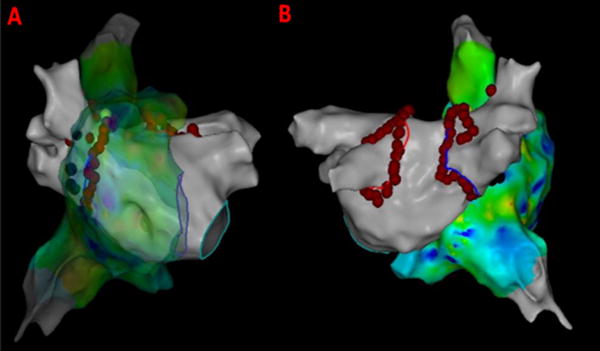
Lesion sets delivered to the persistent AF patient. (A) Antero-Posterior view with RA transparent; (B) Postero-Anterior view; Red dots indicate the lesion points where RF energy was delivered for PVI.

Also, CFAE points were obtained from the CARTO system and superimposed on the LAT map to give insights about atrial electrogram fractionation for comparison with the results from novel 3D maps shown in Figure 2. The CFAE points are defined with a minimum number peaks of 5 and a maximum of 8 in the CARTO system. The red dots in Figure 2 have interval confidence level (ICL) > 8 and the blue dots fit within the 5–8 peak range showing several CFAE positive electrograms throughout LA and RA. In specific, high CFAE regions are seen near RA-LA septum, below left superior pulmonary vein (LSPV) and posterior region of the right sided wide area circumferential ablation (WACA) region have higher CFAE scores indicting the possibility of AF active sites. Figure 3 shows the lesion sets that were delivered to the AF patient to terminate AF.

**Figure 4:**
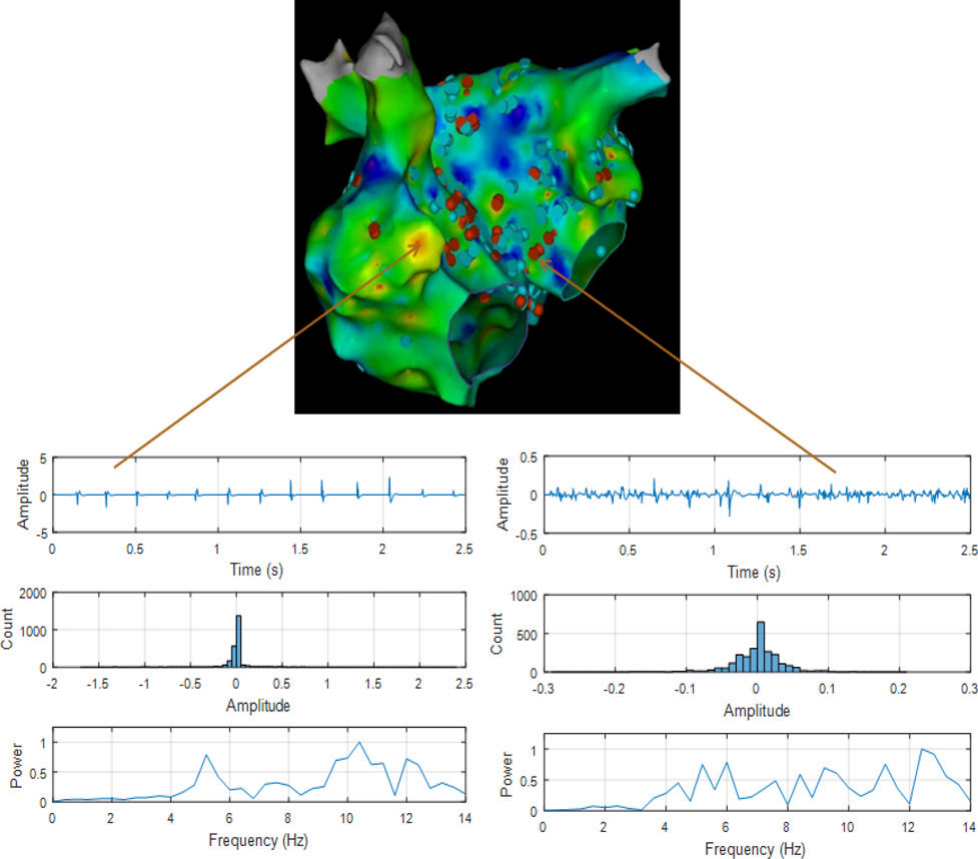
Top-shows 3D LAT map with CFAE points in antero-posterior view. (Bottom-Left) shows a regular electrogram (top panel), its amplitude histogram (middle panel) and power spectrum, (bottom panel). (Bottom-Right) shows an irregular electrogram with high CFAE score (to panel), its amplitude histogram (middle panel) and power spectrum, (bottom panel).

As evident from Figures 1 and 2 there are several non-overlapping regions of low activation and high CAFÉ scores and vice versa which often provides clinical challenges in identifying true activation sites and therefore provides little guidance for the ablation procedure due to the known limitations of these two techniques. However, information from these maps can be used in conjunction from the results with the novel approaches since AF active sites have lower LAT and higher CFAE score, which can be used for comparison for the purposes of this research. Figure 4 shows a regular electrogram from high LAT area and a chaotic electrogram with a high CFAE score indicated by the red dot with their corresponding amplitude histogram and power spectrum demonstrating their temporal and frequency characteristics.

## Results

**Figure 5:**
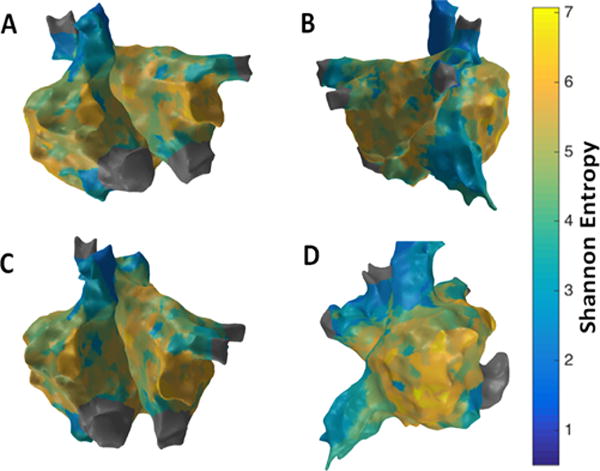
3D Shannon Entropy (SE) map; (A) Antero-Posterior view; (B) Postero-Anterior view; (C) Left Anterior Oblique (LAO) view; (D) Right Anterior Oblique (RAO) view

Figure 5 shows the patient-specific 3D SE map in four different views and higher SE regions are seen at the RAA and the lateral wall seems to have the highest value indicating the possibility of active sites in these regions. However, no specific conclusions can be made on rotor identification. There is some correlation with the LAT map with low activity near RAA where higher SE is seen. However, comparison with CFAE map implies no significant overlap of high fractionation areas with higher SE regions.

**Figure 6:**
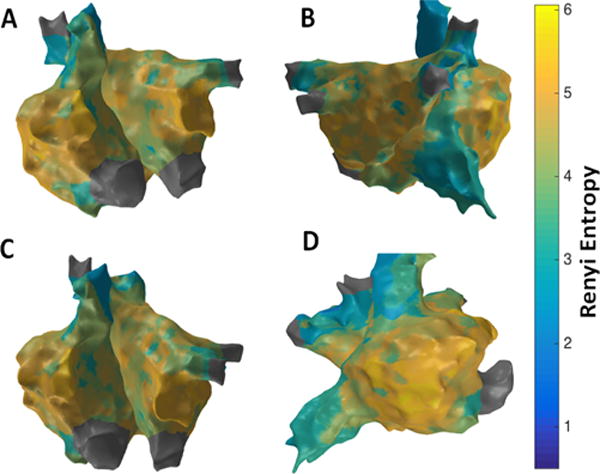
3D Renyi Entropy (RE) map; (A) Antero-Posterior view; (B) Postero-Anterior view; (C) Left Anterior Oblique (LAO) view; (D) Right Anterior Oblique (RAO) view

Figure 6 shows the patient-specific 3D RE map in four different views. Similar to the 3D SE map, higher RE regions are seen at the RAA, lateral wall and right side of the roof seems to have the highest RE value indicating the possibility of active sites in these regions.

**Figure 7:**
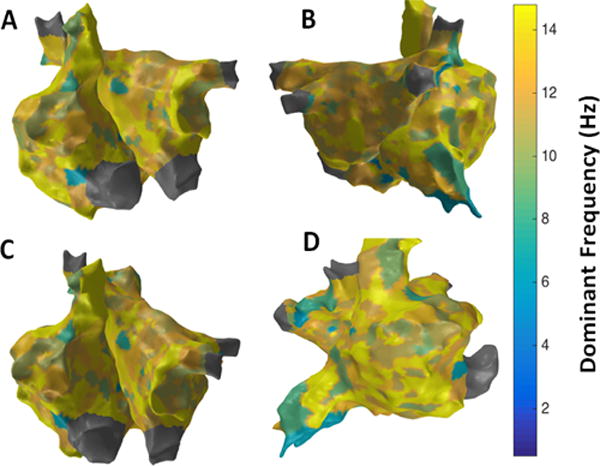
3D Dominant Frequency (DF) map; (A) Antero-Posterior view; (B) Postero-Anterior view; (C) Left Anterior Oblique (LAO) view; (D) Right Anterior Oblique (RAO) view

Figure 7 shows the patient-specific 3D DF map in four different views. More or less uniform DF region is observed with few focal areas of high and low DF in particular high DF region observed in lateral part of RA.

**Figure 8:**
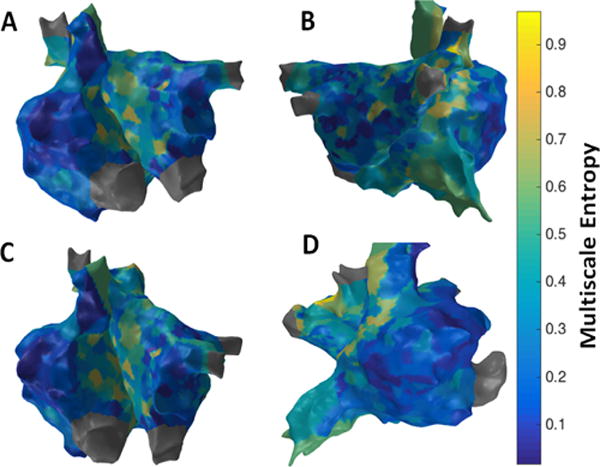
3D Multiscale Entropy (MSE) map; (A) Antero-Posterior view; (B) Postero-Anterior view; (C) Left Anterior Oblique (LAO) view; (D) Right Anterior Oblique (RAO) view

Figure 8 shows the patient-specific 3D MSE map with τ =3 in four different views. MSE approach suggests more localized areas of higher MSE particularly in the region of the septum near the right sided pulmonary veins, posterior aspect of the right veins and posterior wall. The right sided septum, due to far field LA signals, however may reflect a false positive. The high density of MSE in the area of the right veins suggests a high possibility of active site.

**Figure 9:**
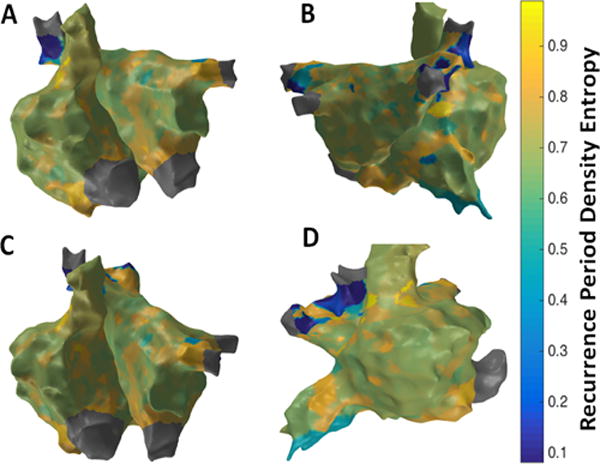
3D Recurrence Period Density Entropy (RPDE) map; (A) Antero-Posterior view; (B) Postero-Anterior view; (C) Left Anterior Oblique (LAO) view; (D) Right Anterior Oblique (RAO) view

Figure 9 shows the patient-specific 3D RPDE map in four different views. RPDE approach suggests more localized areas of higher RPDE particularly in the region of the septum near the right sided pulmonary veins, superior vena cava (SVC), lateral RA and anterior region of inferior vena cava (IVC). Similar to the results from 3D MSE map, 3D RPDE map shows focal areas at the right sided septum, and around SVC and IVC regions suggesting potentially active sites.

**Figure 10:**
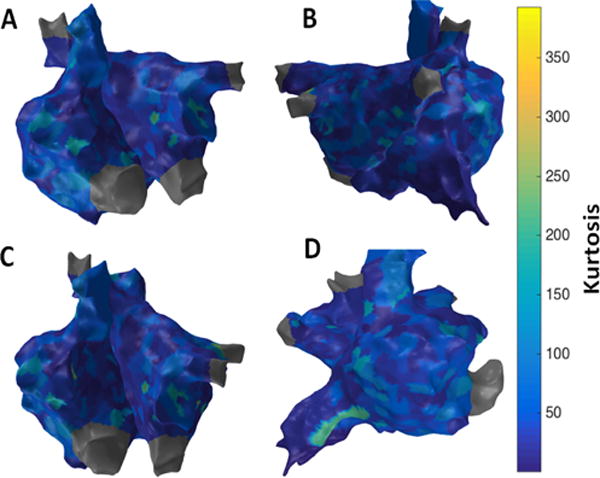
3D Kurtosis map; (A) Antero-Posterior view; (B) Postero-Anterior view; (C) Left Anterior Oblique (LAO) view; (D) Right Anterior Oblique (RAO) view

Figure 10 shows the patient-specific 3D kurtosis map in four different views. Kurtosis approach suggests more localized areas of higher kurtosis particularly at anterior regions of IVC, SVC, right sided septum and right sided pulmonary veins suggesting potential active sites.

**Figure 11:**
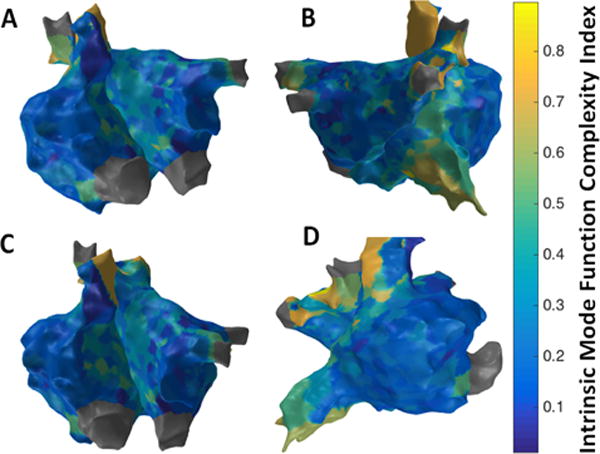
3D Intrinsic Mode Function (IMF) Complexity Index map; (A) Antero-Posterior view; (B) Postero-Anterior view; (C) Left Anterior Oblique (LAO) view; (D) Right Anterior Oblique (RAO) view

Figure 11 shows the patient-specific 3D IMF complexity map in four different views. IMF complexity using EMD approach suggests more localized areas of higher complexity particularly in the region of the septum near the right sided pulmonary veins, and anterior regions of SVC and IVC. Similar to the results from 3D MSE (Figure 8) and 3D RPDE (Figure 9) map, 3D IMF complexity map in Figure 11 shows focal areas at the right sided septum, and around SVC and IVC regions suggesting potentially active sites.

**Figure 12:**
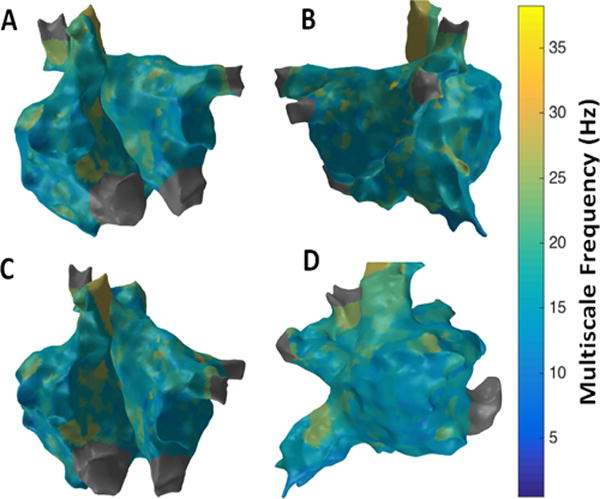
3D Multiscale Frequency (MSF) map; (A) Antero-Posterior view; (B) Postero-Anterior view; (C) Left Anterior Oblique (LAO) view; (D) Right Anterior Oblique (RAO) view

Figure 12 shows the patient-specific 3D MSF map in four different views. MSF approach suggests more localized areas of higher MSF particularly in the region of the septum near the right sided pulmonary veins, superior vena cava (SVC), lateral RA and anterior region of inferior vena cava (IVC). Similar to the results from 3D MSE, 3D RPDE and, 3D IMF complexity maps, 3D MSF map in Figure 12 shows focal areas at the right sided septum, and around SVC and IVC regions suggesting potentially active sites. Overlapping high CFAE, late activation regions and high MSE, RPDE, kurtosis, IMF complexity and MSF can be observed around the RA LA septum and right sided PV regions. However, no specific clinical information can be derived especially on rotor identification or confirm the presence of rotors themselves from these 3D maps.

## Discussion

The feasibility of using conventional catheter mapping system to generate patient-specific 3D maps was demonstrated that can potentially provide near-real time clinical guidance during ablation in clinical setting. Patient specific 3D maps were obtained for a persistent AF patient by processing the electrograms obtained from a pentarray catheter with sequential acquisition using the novel quantitative electrogram analysis approaches such as MSE, MSF, kurtosis, RPDE and EMD. These novel approaches were validated using optical mapping data which was also depolarization data obtained using voltage-sensitive dyes in isolated ex-vivo rabbit heart model of ventricular arrhythmia. Since intracardiac electrograms also represent depolarization data measured from the electrodes, the novel signal processing that was validated using optical mapping depolarization data is translatable for quantitative intracardiac electrogram analysis. However, confirming the existence of rotors in human AF is extremely challenging although several group have demonstrated with their custom methods, there are no known reliable methods, and therefore rotor pivot point identification as suitable ablation targets to terminate AF becomes an open question, which needs several clinical studies to reliably demonstrate the presence of rotors in first place.

The results from the 3D maps from the novel approaches revealed several focal regions of high complexity in the 3D MSE, RPDE, IMF and MSF maps. These maps demonstrated the possibility of active sites at RA LA septum, right sided pulmonary veins and anterior regions of SVC and IVC with high complexity from these various time domain and frequency domain techniques. However, no clinical inferences could be made on rotor identification or even the presence of rotors. Nevertheless, these 3D maps demonstrated the fact that active sites were present outside the PV region for a persistent AF patient which is consistent with the literature. The results also suggest the necessity to use a comprehensive 3D map that takes into account information from these various techniques that can possibly provide a faithful focal region of interest that may be an active site. Validation with several AF patient datasets is required to make clinical inferences on the pattern of complexity regions in these patients to assess whether or not rotors are present and if rotor pivot points were identified using these techniques.

A major limitation of using these novel approaches validated with optical mapping data is that optical data from the animal model is high resolution uniform data and mostly noise free. Intracardiac mapping is achieved with sequential mapping that acquires sparse data throughout the atria with very low resolution and contaminated with various noises such as baseline wander noise, VFF and other high frequency noise. In this study, bipolar electrograms were used which is relatively free from VFF noise compared to unipolar electrograms. However, slight degree of VFF contamination is always possible depending on the spatial location of the mapping catheter. No preprocessing was performed in the intracardiac electrograms to obtain the 3D maps, which might have affected the results to slight extent if indeed VFF noise was present in some of the processed electrograms. Therefore, future work should include VFF noise removal approaches to reduce ambiguity from VFF noise contamination. Nevertheless, these novel approaches offers promise in providing new avenues for electrogram analysis for possible rotor localization with more controlled animal and human studies. These improved methods can further be validated to demonstrate whether or not ablation at these rotor pivot points in humans can essentially terminate AF permanently which can create a paradigm shift in the clinical management of patients with AF.

## Conclusion

This study has demonstrated the feasibility of generating patient specific 3D maps using conventional catheter mapping system using novel quantitative electrogram analysis approaches. Several focal areas of active sites were implied in a persistent AF patient outside the PV region through these different 3D maps, however no clinical inferences could be made. Also, the results with focal regions of higher complexity do not imply the presence of rotor pivot points or the presence of rotors themselves. Rigorous testing with several clinical datasets from both paroxysmal and persistent AF patients is required for validation of these results that may provide new insights into rotor identification using these novel approaches. The results however offer promise to generate patient specific 3D maps that can potentially provide near-real time clinical guidance for AF ablation.

## Abbreviations

AF: Atrial fibrillation
PVI: Pulmonary Vein Isolation
CFAE: Complex Fractionated Electrogram Analysis
MSE: Multiscale Entropy
MSF: Multiscale Frequency
RPDE: Recurrence Period Density Entropy
EMD: Empirical Mode Decomposition
CTI: Cavotricuspid Isthmus
IMF: Intrinsic Mode Functions
RAA: Right Atrial Appendage
IVC: Inferior Vena Cava
WACA: Wide Area Circumferential Ablation
LPSV: Left Superior Pulmonary Vein
